# Development of a Sliding-Mode-Control-Based Path-Tracking Algorithm with Model-Free Adaptive Feedback Action for Autonomous Vehicles

**DOI:** 10.3390/s23010405

**Published:** 2022-12-30

**Authors:** Kwangseok Oh, Jaho Seo

**Affiliations:** 1 School of ICT, Robotics & Mechanical Engineering, Hankyong National University, Anseong-si 17579, Republic of Korea; 2 Department of Automotive and Mechatronics Engineering, Ontario Tech University, Oshawa, ON L1G 0C5, Canada

**Keywords:** model-free adaptive feedback, sliding mode control, path tracking, autonomous vehicle, recursive least squares, forgetting factor, Lyapunov stability

## Abstract

This paper presents a sliding mode control (SMC)-based path-tracking algorithm for autonomous vehicles by considering model-free adaptive feedback actions. In autonomous vehicles, safe path tracking requires adaptive and robust control algorithms because driving environment and vehicle conditions vary in real time. In this study, the SMC was adopted as a robust control method to adjust the switching gain, taking into account the sliding surface and unknown uncertainty to make the control error zero. The sliding surface can be designed mathematically, but it is difficult to express the unknown uncertainty mathematically. Information of priori bounded uncertainties is needed to obtain closed-loop stability of the control system, and the unknown uncertainty can vary with changes in internal and external factors. In the literature, ongoing efforts have been made to overcome the limitation of losing control stability due to unknown uncertainty. This study proposes an integrated method of adaptive feedback control (AFC) and SMC that can adjust a bounded uncertainty. Some illustrative and representative examples, such as autonomous driving scenarios, are also provided to show the main properties of the designed integrated controller. The examples show superior control performance, and it is expected that the integrated controller could be widely used for the path-tracking algorithms of autonomous vehicles.

## 1. Introduction

In addition to advanced hardware components such as steering, braking, and driving components, autonomous driving technology is one of the most important mobility technologies for improving safety, efficiency, and convenience. Because an autonomous vehicle aims to drive under any driving conditions and environment by itself, it needs various sensors—such as cameras, LiDAR, radar, and ultrasonic sensors—that can replace human sensory organs. In addition, mechanical actuators such as electric or hydraulic motors that can replace human muscle are needed to produce the desired force or pressure. Moreover, a computing system that functions like a human brain is required for data processing and decision-making for autonomous driving. Consequently, the vehicle system is more complicated and nonlinear as a result of the necessity of these various components that allow it to perform various driving tasks—such as lane changing, automatic parking, car-following, etc.

For driving tasks, accurate path-tracking performance should be ensured with reasonable path planning. Because vehicle conditions and driving conditions/environments can change unexpectedly, the path-tracking performance of autonomous vehicles can be degraded, causing fatal accidents on the road. To overcome the aforementioned limitation, various control technologies for the path tracking of autonomous vehicles have been developed, as follows.

### 1.1. Literature Review

Sun, C. et al. presented a model predictive control (MPC) path-tracking controller with switched tracking errors that can reduce the lateral tracking deviation and maintain vehicle stability for both normal and high-speed conditions [1]. They compared the performance of three MPC controllers with different tracking errors and analyzed their results. Baca, T. et al. proposed a linear MPC-based novel approach for optimal trajectory tracking for unmanned aerial vehicles (UAVs) using nonlinear state feedback [2]. They demonstrated the usability of the proposed approach through statistical and experimental evaluations of the platform in both simulated and real-world examples. Suh, J. et al. developed motion-planning algorithms for lane changing with a combination of probabilistic and deterministic prediction methods for automated driving under complex driving circumstances [3]. A collision probability and a safe driving envelope were defined by the authors using a reachable set and behavioral prediction of surrounding vehicles for safe lane changing. The developed model was evaluated based on simulations and experiments using an actual test vehicle under a lane change scenario. Xu, S. and Peng, H. presented a preview steering control algorithm for accurate, smooth, and computationally inexpensive path tracking for automated vehicles, along with an analysis of the closed-loop system [4]. In the study, the future road curvature as a dynamic disturbance was considered for the preview controller design, and its performance was evaluated based on simulations and experimental tests. Chowdhri, N. et al. developed a nonlinear MPC algorithm to perform evasive maneuvers and avoid a rear-end collision, with constraints [5] that are needed for ensuring vehicle stability and accounting for actuator limitations. Li, S. et al. proposed an obstacle avoidance controller based on nonlinear MPC for autonomous vehicle navigation [6]. It was designed so that the reference trajectory is adjusted when obstacles suddenly appear and the risk index is computed online for collision avoidance. Cao, J et al. developed a trajectory-tracking control algorithm for autonomous vehicles considering cornering characteristics with simplified vehicle dynamics and tire models [7]. Wang, Y et al. developed an MPC algorithm to optimize the reference trajectory with consideration of the motion prediction of other traffic participants using Monte Carlo simulations [8]. Quirynen, R. et al. studied the real-time feasibility of nonlinear MPC-based steering control on an embedded computer for autonomous vehicles [9]. In addition, Shen, C and Shi, Y investigated the nonlinear model predictive control (NMPC) method, looking for possible approaches to alleviate the heavy computational burden, and developed novel distributed NMPC algorithms by exploiting the dynamic properties of the autonomous underwater vehicle motion for trajectory-tracking control [10]. Chu, D. et al. presented a trajectory planning and tracking framework to obtain target trajectory and MPC with PID feedback to effectively track planned trajectory [11]. In [12], an improved MPC algorithm with fuzzy adaptive weight control was proposed for autonomous vehicles to ensure tracking accuracy and dynamic stability during path tracking. To implement trace planning and tracking for obstacle avoidance, Zhang, C et al. integrated a trajectory planner and a tracking controller for autonomous vehicles [13]. The study of [14] proposed a scheme for implementing an MPC path-following controller that considers feasible road regions, vehicle shapes, and the model mismatch caused by varying road conditions and small-angle assumptions in measurable disturbances [14]. To maintain a collision-free path for autonomous vehicles, the authors of [15] proposed a hierarchical path-planning and trajectory-tracking framework by solving a constrained finite-time optimal problem. Yue, M et al. developed a time-based quantic polynomial function for trajectory planning that takes into account the vehicle system’s safety, comfort, and traffic efficiency [16]. A robust MPC with a finite time horizon was proposed by Peng, H et al. to achieve coordinated path tracking and direct yaw moment control for autonomous four-in-wheel-motor independent-drive electric vehicles [17].

The previous studies mentioned above used mathematical vehicle models to design path-tracking control algorithms; however, there are model uncertainties that have a negative impact on the path-tracking control performance. Hence, studies on the adaptive path-tracking control of autonomous vehicles have been conducted to reduce model uncertainty and improve performance under various driving conditions and environments.

Londhe, P. and Patre, B. designed a robust and adaptive tracking control algorithm for a complete nonlinear model of an autonomous underwater vehicle based on adaptive fuzzy sliding mode control (SMC) [18]. The study derived fuzzy control rules using the Lyapunov energy function to minimize chattering. Taghavifar, H. and Rakheja, S. applied an exponential-like sliding mode fuzzy type-2 neural network approach to design a robust adaptive indirect controller that can enhance the path-tracking performance of autonomous road vehicles [19]. In this study, the authors used the Lyapunov stability theorem to derive the adaptation laws for a hierarchical controller design and ensure the stability of the closed-loop system.

Zhou, X. et al. proposed an adaptive inverse controller to offset the dynamics of the steering system’s backlash, and adaptive control laws were robustified by means of sigma modification [20]. The authors presented hardware-in-the-loop experimental results to show the main contribution of the proposed control algorithm. Yuan, X. et al. developed a course-angle optimal referential model and MPC-based adaptive control system for more adaptive path tracking at different velocities [21].

To improve tracking accuracy and stability, Lin, F. et al. developed an adaptive MPC controller by applying a recursive least squares algorithm that can estimate cornering stiffness and road friction online [22]. Liu, S et al. proposed a novel model-free adaptive control algorithm based on a dual successive projection method and analyzed it using the introduced method with a symmetrically similar structure of the controller [23]. Guerrero, J et al. designed an adaptive high-order sliding mode controller that does not require knowledge of the upper bound of the disturbance for trajectory tracking with the Lyapunov concept [24]. Tran, V et al. proposed a new concept of an adaptive strictly negative imaginary controller that minimizes a certain performance index robustly for 3D tracking of drones in the face of wind gusts [25]. Tian, Y et al. developed an adaptive path-tracking control strategy that coordinates active front steering and direct yaw moment based on an MPC algorithm. The authors used the recursive least squares method with a forgetting factor to identify the rear tires’ cornering stiffness and update the path-tracking system prediction model [26]. For robust adaptive path tracking of an underactuated unmanned surface vehicle, Fan, Y et al. proposed an improved line-of-sight guidance law using a reduced-order extended state observer to address the large sideslip angle that occurs in practical navigation. [27]. Pereida, K and Schoellig, A developed a novel adaptive MPC with an underlying L_1_ adaptive controller to enhance the trajectory tracking of a system under unknown and changing disturbances [28]. Kebbati, Y et al. presented an improved particle-swarm-optimized PID to handle the task of speed tracking based on nonlinear longitudinal dynamics for the coordinated longitudinal and lateral control in autonomous driving [29]. By applying dynamic trajectory planning and a robust adaptive nonlinear fuzzy backstepping controller, a novel nonlinear trajectory-tracking control strategy was developed for lane-changing maneuvers [30]. A sliding mode control approach with enhanced state observers was proposed in [31] to control both lane-keeping errors and roll angles within the prescribed performance boundaries. Liang, Y et al. proposed a novel scheme that integrates local motion planning and control to determine motion behaviors, track global paths, and conduct local motion commands based on adaptive MPC and lateral MPC [32]. For autonomous vehicles with four independent in-wheel motors, an integrated autonomous driving (AD) control system was developed in [33], consisting of two parts: an AD controller and a chassis controller. He, H et al. presented a hierarchical path-tracking control framework for two-axle autonomous buses with two layers that can prevent sideslip and rollover and can acquire the steering angle with stability constraints [34]. In order to design adaptive control algorithms for path tracking, mode-based or model-free adaptation rules are needed for control input adaptation. However, it is difficult to design adaptation rules ensuring robust stability of control systems while taking constraints into account. To tackle this issue, data-driven or learning-based path-tracking control algorithms have been developed.

Chen, I. and Chan, C. developed deep reinforcement learning algorithms using proximal policy optimization that were combined with the conventional pure pursuit method to structure the controller’s architecture [35]. Zhang, K. et al. proposed an adaptive learning MPC scheme for the trajectory tracking of perturbed autonomous ground vehicles based on unknown system parameter estimation [36]. The authors designed a set-membership-based parameter estimator using the recursive least squares technique. Jiang, Y. et al. investigated the path tracking control strategy of variable-configuration unmanned ground vehicle and proposed an improved model free predictive control scheme [37]. Li, X. et al. developed a novel robust adaptive learning control algorithm that can estimate the system uncertainties through the iterative learning method [38]. In this design, a two-degree-of-freedom vehicle model was reformulated into a parametric form. Wang, Z and Wang, J incorporated model-free strategies for control and direct data-driven control into a predictive control framework for trajectory tracking of automated vehicles [39]. For unmanned surface vehicles, Wang, N et al. developed an innovative self-learning system using only input–output signals [40]. They developed a data-driven performance-prescribed reinforcement learning control scheme to pursue control optimality and prescribe tracking accuracy simultaneously. Jiang, Y et al. studied the heading tracking problem of six-wheel independent-drive and four-wheel independent-steering unmanned ground vehicles under the influence of uncertainties based on the model-free adaptive control method and particle swarm optimization [41]. Parseh, M et al. proposed a data-driven motion planning method to minimize injury severity for vehicle occupants in unavoidable collisions by establishing a metric that models the relationship between impact location and injury severity using real accident data [42]. Wu, Q et al. developed a fuzzy-inference-based reinforcement learning approach for autonomous overtaking decision-making that was created using a multi-objective Markov decision process and a temporal difference learning method based on dynamic fuzzy inference [43]. By integrating model-free control and extreme-seeking control, Wang, Z et al. provided a new perspective on tuning model-free control gain while improving its performance [44]. Spielberg, N et al. designed a neural network MPC using vehicle operation data to construct a neural network model that could be efficiently implemented in MPC [45]. Peng, Z et al. proposed reduced- and full-order data-driven adaptive disturbance observers for estimating unknown input gains, as well as total disturbances consisting of unknown internal dynamics and external disturbances [46]. To avoid collisions efficiently, Wang, H and Liu, B proposed a collision-avoidance framework based on road friction estimation and dynamic stability control [47]. The study of [48] aimed to develop a model-based feasibility enhancement technique of constrained reinforcement learning that can enhance the feasibility of policies using a generalized control barrier function that is defined based on the distance to the constraint boundary [48]. With an iterative single-critic learning framework, Zhang, K et al. proposed adaptive resilient event-triggered control for rear-wheel-drive autonomous vehicles [49]. This control can be effective in balancing frequency and changes when adjusting the vehicle’s control during the running process. Combining the event-triggered sampling mechanism and the iterative single-critic learning framework, the authors developed an event-triggered condition for adaptive resilient control.

### 1.2. Summary of the Proposed Control Algorithm and Major Contributions

Suitable path-tracking performance is essential for the driving tasks of autonomous vehicles, such as lane changing, automatic parking, and vehicle following. However, path-tracking performance can be degraded by unexpected and abrupt changes in vehicle conditions and the driving environment. To deal with this issue and ensure robust control performance, our study designed a new path-tracking control algorithm by integrating adaptive feedback control (AFC) inputs with SMC. Specifically, the AFC algorithm was created using the recursive least squares and gradient descent methods to adjust feedback gains. It was designed so that the SMC algorithm was able to consider the error terms regulated by the AFC input with finite stability and Lyapunov stability conditions. Furthermore, the designed SMC algorithm is capable of considering the error terms regulated by the AFC input with finite stability and Lyapunov stability.

The performance evaluation of the proposed path-tracking control algorithm was conducted under two scenarios: curved path tracking, and lane change scenarios with constant velocity conditions.

The following is a summary of the major contributions of this study:

- The proposed control method is an attempt to develop an integrative control algorithm for path tracking of autonomous vehicles using adaptive feedback and SMC algorithms that can reject model uncertainties and ensure robust stability.
- The proposed control scheme allows for the design of controllers using a simple mathematical model that requires low computational costs.

Based on the literature review above, Table 1 summarizes the pros and cons of the proposed control method in comparison with other related existing approaches, which are classified into five categories.

The remainder of this paper is outlined as follows: Section 2 presents a control algorithm for path tracking using SMC with adaptive feedback. Section 3 provides the results of the performance evaluation. Section 4 concludes with a discussion of the limitations of the current work and prospects for future research.

## 2. SMC-Based Path Tracking with Adaptive Feedback Action

**Figure 1:**
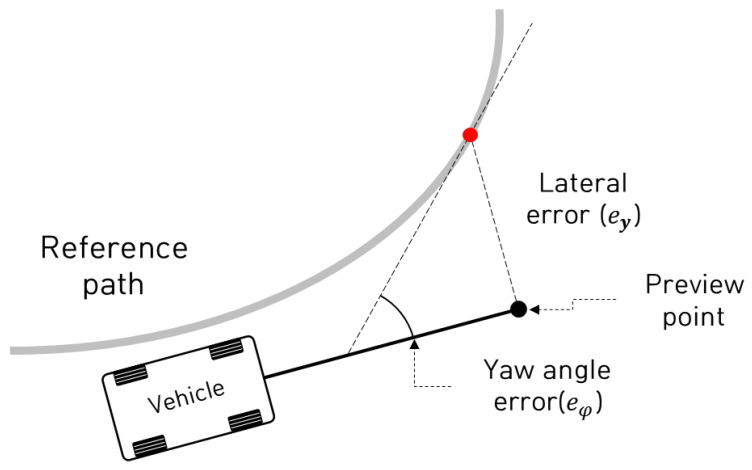
Defined control errors for path tracking.

This section provides the mathematical formulation of the SMC-based path-tracking algorithm with adaptive feedback action. In order to design the path-tracking control algorithm, a kinematic mathematical error model was used. Figure 1 shows defined control errors such as lateral error and yaw angle error for path tracking.

**Figure 2:**
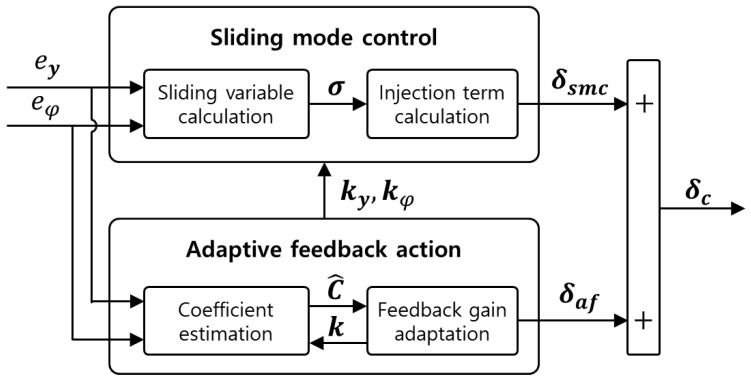
Block diagram for the adaptive feedback action-based sliding mode control.

Based on the defined path-tracking error, a kinematic-analysis-based mathematical error model was derived. The following equations represent the mathematical error model using kinematic parameters and its state-space representation:

(1) $${\dot{e}}_{y}=v_{x}e_{\varphi }$$

(2) $${\dot{e}}_{\varphi }=\frac{v_{x}}{L}\delta -{\dot{\varphi }}_{d}$$

(3) $$\left[\begin{array}{c} {\dot{e}}_{y}\\ {\dot{e}}_{\varphi } \end{array}\right]=\left[\begin{array}{cc} 0 & v_{x}\\ 0 & 0 \end{array}\right]\left[\begin{array}{c} e_{y}\\ e_{\varphi } \end{array}\right]+\left[\begin{array}{c} 0\\ v_{x}/L \end{array}\right]\delta +\left[\begin{array}{c} 0\\ -1 \end{array}\right]{\dot{\varphi }}_{d}$$

where $e_{y}$ and $e_{\varphi }$ are the lateral error and yaw angle error with respect to the reference path for tracking of an autonomous vehicle, respectively, while $v_{x}$, ${\dot{\varphi }}_{d}$, δ, and L are the longitudinal velocity, desired yaw rate, front steering angle, and wheel base (i.e., the distance between the front-wheel axle and rear-wheel axle) of the vehicle, respectively. Figure 2 shows an overall block diagram for the model-free adaptive feedback action-based SMC algorithm.

The coefficient for feedback gain adaptation (the coefficient estimation block under the adaptive feedback action in Figure 2) can be estimated using the recursive least squares method with a forgetting factor. Using the estimated coefficient, a feedback gain is adapted based on the gradient descent method with a proper adaptation gain. The adaptive steering control input is calculated using the adapted feedback gain and the path-tracking control error. In this study, the SMC input for path tracking was computed with consideration of the adaptive steering control input to reduce the impact of the SMC input on the path-tracking control performance. The following equations were used to calculate the total steering control input using adaptive and sliding control inputs. In addition, mathematical definitions of the adaptive steering control and SMC inputs are presented below:

(4) $$\delta_{c}=\delta_{af}+\delta_{smc}$$

(5) $$\delta_{af}=k_{y}e_{y}+k_{\varphi }e_{\varphi }$$

(6) $$\delta_{smc}=-\rho \mathrm{sign}\left(\sigma \right)$$

where $\delta_{c}$ is the total control input for the front steering wheel angle, $\delta_{af}$ and $\delta_{smc}$ are the adaptive feedback-based control input and SMC-based control input, respectively, $k_{y}$ and $k_{\varphi }$ are the feedback gains for the lateral and yaw angle errors, respectively, and ρ and σ are the magnitudes of the SMC input and sliding surface for controller design, respectively. Equation (3) can be rewritten by using the AFC input described in Equation (5). The following state-space-formed error mathematical model is the rewritten equation of Equation (3) using Equation (5):

(7) $$\left[\begin{array}{c} {\dot{e}}_{y}\\ {\dot{e}}_{\varphi } \end{array}\right]=\left[\begin{array}{cc} 0 & v_{x}\\ k_{y}v_{x}/L & k_{\varphi }v_{x}/L \end{array}\right]\left[\begin{array}{c} e_{y}\\ e_{\varphi } \end{array}\right]+\left[\begin{array}{c} 0\\ v_{x}/L \end{array}\right]\delta_{smc}+\left[\begin{array}{c} 0\\ -1 \end{array}\right]{\dot{\varphi }}_{d}$$

In this study, the SMC input for path tracking was computed based on Equation (7). Calculating SMC inputs requires information about adaptive feedback gains, whose adaptation algorithms are explained in the next section.

### 2.1. Adaptive Feedback Action for Feedback Gain Adaptation

To estimate the coefficients for feedback gain adaptation, the two relationship functions shown in Equation (8) were designed and used for recursive least squares estimation with forgetting factors. This equation relates control errors to feedback gains for the derivation of coefficients $C_{\mathrm{ij}}\left(i,j=1,2\right)$ [50].

(8) $${\dot{e}}_{y}=C_{11}{\dot{k}}_{y}+C_{12}{\dot{k}}_{\varphi }\;{\dot{e}}_{\varphi }=C_{21}{\dot{k}}_{y}+C_{22}{\dot{k}}_{\varphi }$$

The coefficients are estimated based on recursive least squares with properly determined forgetting factors, which are used for the feedback gain adaptation. The feedback gain is adapted by using the gradient descent method to minimize the control errors. The following equation is the cost function $J_{af}$ defined for the gradient descent method:

(9) $$J_{af}=\frac{1}{2}e_{y}^{2}+\frac{1}{2}we_{\varphi }^{2}$$

Based on the gradient descent method with the cost function defined above, the following feedback gain adaptation rules can be derived to reduce the control errors using the adaptation gain, weighting factor, and partial derivatives of path-tracking control errors with respect to feedback gains:

(10) $${\dot{k}}_{y}=-\gamma_{y}\frac{\partial J_{af}}{\partial k_{y}}=-\gamma_{y}\left(e_{y}+we_{\varphi }\right)\left(\frac{\partial e_{y}}{\partial k_{y}}+w\frac{\partial e_{\varphi }}{\partial k_{y}}\right)$$

(11) $${\dot{k}}_{\varphi }=-\gamma_{\varphi }\frac{\partial J_{af}}{\partial k_{\varphi }}=-\gamma_{\varphi }\left(e_{y}+we_{\varphi }\right)\left(\frac{\partial e_{y}}{\partial k_{\varphi }}+w\frac{\partial e_{\varphi }}{\partial k_{\varphi }}\right)$$

In this study, it was assumed that the estimated coefficients in Equation (8) were approximately equal to the partial derivatives of the path-tracking errors with respect to the feedback gains. Because this assumption may lead to unexpected control uncertainty, it was designed so that the SMC algorithm featured AFC inputs to ensure robustness. The following Equations (12) and (13) are rewritten versions of Equations (10) and (11) with this assumption; Equation (14) is the detailed AFC input obtained using the adapted feedback gains and adaptation gains:

(12) $${\dot{k}}_{y}=-\gamma_{y}\frac{\partial J_{af}}{\partial k_{y}}=-\gamma_{y}\left(e_{y}+we_{\varphi }\right)\left({\hat{C}}_{11}+w{\hat{C}}_{21}\right)$$

(13) $${\dot{k}}_{\varphi }=-\gamma_{\varphi }\frac{\partial J_{af}}{\partial k_{\varphi }}=-\gamma_{\varphi }\left(e_{y}+we_{\varphi }\right)\left({\hat{C}}_{12}+w{\hat{C}}_{22}\right)$$

(14) $$\delta_{af}=-e_{y}\int \gamma_{y}\left(e_{y}+we_{\varphi }\right)\left({\hat{C}}_{11}+w{\hat{C}}_{21}\right)dt\;\;-e_{\varphi }\int \gamma_{\varphi }\left(e_{y}+we_{\varphi }\right)\left({\hat{C}}_{12}+w{\hat{C}}_{22}\right)dt$$

The next subsection explains the SMC algorithm that considers the designed AFC input for robust path-tracking performance of autonomous vehicles.

### 2.2. SMC with Adaptive Feedback Action

The AFC algorithm described in the previous subsection can adapt the feedback gain to reduce the path-tracking control, but it cannot guarantee the stability of the control algorithm if it is used alone. Therefore, an SMC algorithm that can consider the adaptation influence on the path-tracking performance is proposed in this study, based on the integration of two control algorithms (such as adaptive feedback and robust control algorithms).

By integrating the adaptive feedback and robust control algorithms, uncertainties can be reduced by the feedback gain adaptation, while stability can be ensured by the robustness of the sliding mode controller. In this study, a sliding surface (σ) was designed for path tracking using the following equation:

(15) $$\sigma =e_{y}+we_{\varphi }$$

where w is the weighting factor for the design of a sliding surface. The following equation is the cost function for the design of the SMC algorithm; the time derivative of the cost function is described in Equation (18) for the control input derivation:

(16) $$J_{smc}=\frac{1}{2}\sigma^{2}$$

(17) $${\dot{J}}_{smc}=\sigma \dot{\sigma }=\sigma \left({\dot{e}}_{y}+w{\dot{e}}_{\varphi }\right)$$

Equation (17) above can be rewritten as follows by applying Equation (7) to derive the SMC input considering the adaptive steering control input:

(18) $${\dot{J}}_{smc}=\sigma \left(v_{x}e_{\varphi }+\frac{wk_{y}v_{x}}{L}e_{y}+\frac{wk_{\varphi }v_{x}}{L}e_{\varphi }+\frac{wv_{x}}{L}\delta_{smc}-w{\dot{\varphi }}_{d}\right)$$

All of the terms in the parentheses of Equation (18)—except for the control input term $\delta_{smc}$—can be considered as disturbances, and an inequality condition using the disturbance boundary value $L_{b}$ can be derived as follows:

(19) $$L_{b}\geq \left|v_{x}e_{\varphi }+\frac{wk_{y}v_{x}}{L}e_{y}+\frac{wk_{\varphi }v_{x}}{L}e_{\varphi }-w{\dot{\varphi }}_{d}\right|$$

In order to design an asymptotically stable controller, the discrete injection term of SMC is defined as follows:

(20) $$\frac{wv_{x}}{L}\delta_{smc}=-\rho \mathrm{sign}\left(\sigma \right)$$

where ρ is the magnitude of the injection term, which was designed by considering the boundary value in Equation (19) for the stability of the controller. Equation (18) can be rewritten as follows using the boundary value and the definition in Equation (20):

(21) $${\dot{J}}_{smc}\leq \sigma \left(L_{b}-\rho \mathrm{sign}\left(\sigma \right)\right)=-\left|\sigma \right|\left(\rho -L_{b}\right)$$

For the finite stability condition, the following inequality condition was derived based on the cost function condition, and the magnitude of the injection term can be determined with Equations (21) and (22):

(22) $${\dot{J}}_{smc}\leq -\left|\sigma \right|\alpha /\sqrt{2}$$

(23) $$\rho =L_{b}+\alpha /\sqrt{2}$$

where α is a parameter for the finite stability condition. Based on the detailed disturbance boundary value, the magnitude of the injection term can be rewritten as follows:

(24) $$\rho =\left|v_{x}e_{\varphi }+\frac{wk_{y}v_{x}}{L}e_{y}+\frac{wk_{\varphi }v_{x}}{L}e_{\varphi }-w{\dot{\varphi }}_{d}\right|+\alpha /\sqrt{2}$$

It is assumed in this study that the AFC input can reduce the control errors reasonably with the SMC input; therefore, the path-tracking control errors $e_{y}$ and $e_{\varphi }$ are taken to be zero. Equation (24) can be simplified based on this assumption, as shown in Equation (25).

(25) $$\rho =\left|w{\dot{\varphi }}_{d}\right|+\alpha /\sqrt{2}$$

Using the magnitude of the injection term ρ in Equation (25), the SMC input can be computed using Equation (20) as follows:

(26) $$\delta_{smc}=-\frac{L}{wv_{x}}\left(\left|w{\dot{\varphi }}_{d}\right|+\alpha /\sqrt{2}\right)\mathrm{sign}\left(\sigma \right)$$

To reduce chattering of the SMC input, a sigmoid function was adopted and used in Equation (26) instead of a sign function. The following equation is the sigmoid-function-based SMC input:

(27) $$\delta_{smc}=-\frac{L}{wv_{x}}\left(\left|w{\dot{\varphi }}_{d}\right|+\alpha /\sqrt{2}\right)\left(\frac{m\sigma }{1+m\left|\sigma \right|}\right)$$

where m is a coefficient that is used to adjust the gradient of the sigmoid function near zero. 

Using Equations (4), (14) and (27), the total steering control input that requires the adaptation gain, weighting factor, and other parameters (α,m) can be derived as follows:

(28) $$\delta_{c}=-e_{y}\int \gamma_{y}\left(e_{y}+we_{\varphi }\right)\left({\hat{C}}_{11}+w{\hat{C}}_{21}\right)dt\;\;-e_{\varphi }\int \gamma_{\varphi }\left(e_{y}+we_{\varphi }\right)\left({\hat{C}}_{12}+w{\hat{C}}_{22}\right)dt\;\;-\frac{L}{wv_{x}}\left(\left|w{\dot{\varphi }}_{d}\right|+\alpha /\sqrt{2}\right)\left(\frac{m\sigma }{1+m\left|\sigma \right|}\right)$$

The next section provides the performance evaluation results under various evaluation scenarios (i.e., curved path tracking and lane change).

## 3. Performance Evaluation

The performance evaluation was conducted using a planar vehicle model called the bicycle model under two path-tracking scenarios: curved path tracking, and lane change. The longitudinal velocities for the curved path tracking and lane change scenarios were kept constant at 30 kph and 60 kph, respectively.

**Figure 3:**
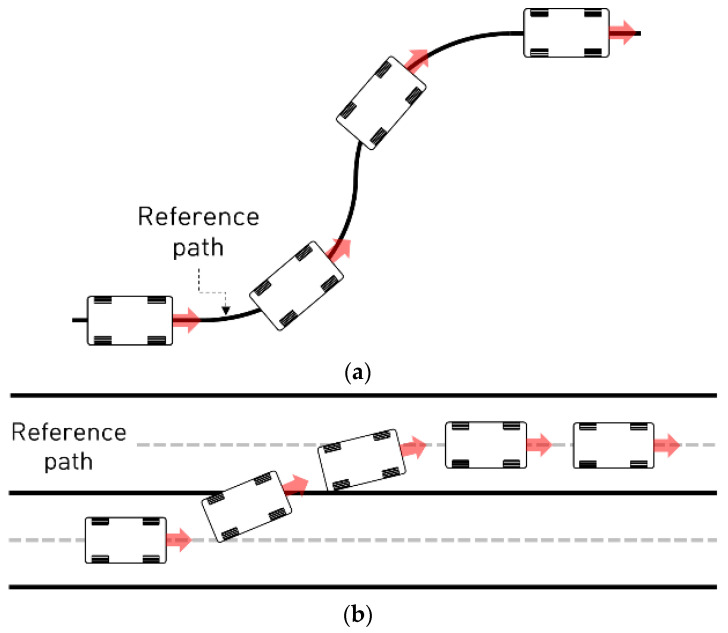
Two evaluation scenarios for performance evaluation: (**a**) Curved path-tracking scenario. (**b**) Lane change scenario.

**Figure 4:**
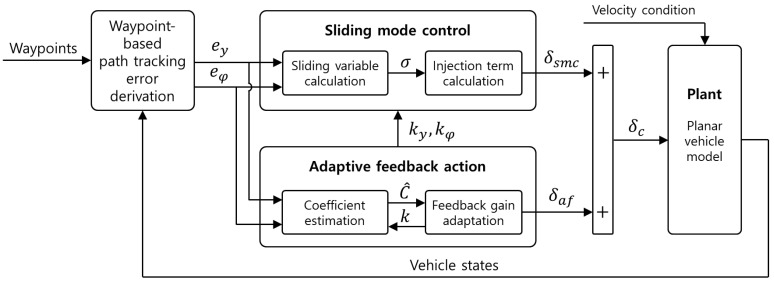
Overall block diagram for performance evaluation of the control algorithm.

For a comparative study, the performance of the different types of designed path-tracking controllers was evaluated four times for each scenario. The control algorithms proposed in this study were designed and evaluated using MATLAB/Simulink. Figure 3 and Figure 4 illustrate the two scenarios and an overall block diagram for the performance evaluation of the designed control algorithm, respectively.

In the waypoint-based path-tracking error derivation block, path-tracking control errors are computed using the designed waypoints and vehicle states in the block. The waypoints consist of *x* and *y* points of reference paths for curved and lane-change paths. Table 2 and Table 3 show the vehicle specifications and the designed control parameters used for the performance evaluation.

The next two subsections show the performance evaluation results for the curved path and lane change scenarios.

### 3.1. Path-Tracking Scenario: Curved Path Tracking (30 kph)

The results were compared between cases using AFC alone, SMC alone, SMC with AFC, and proportional–integral–derivative (PID) control.

**Figure 5:**
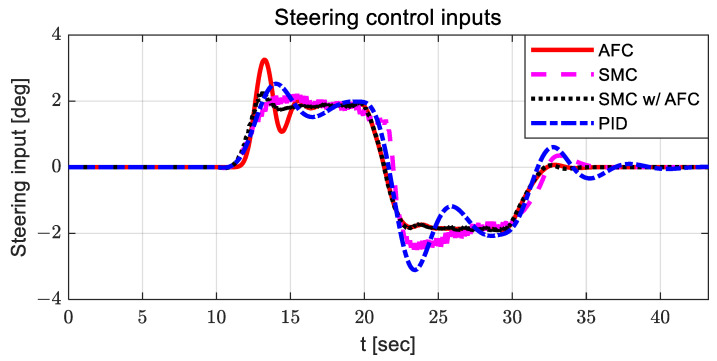
Results: steering control inputs for the curved path tracking.

The radius of curvature of the designed curved path was 100 m, and the longitudinal velocity of the vehicle was 30 kph. Figure 5 shows the steering control inputs for path tracking of all evaluation cases.

**Figure 6:**
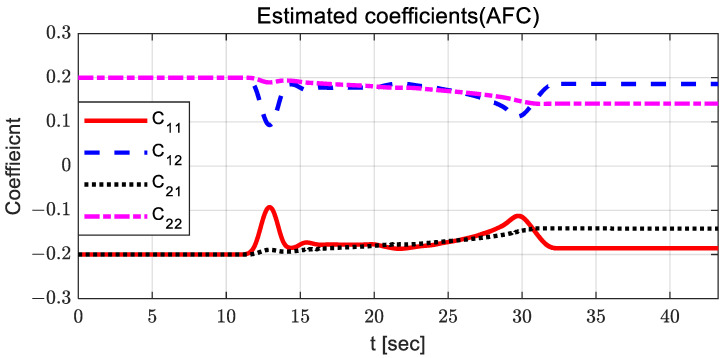
Results: estimated coefficients in the case of AFC for the curved path tracking.

**Figure 7:**
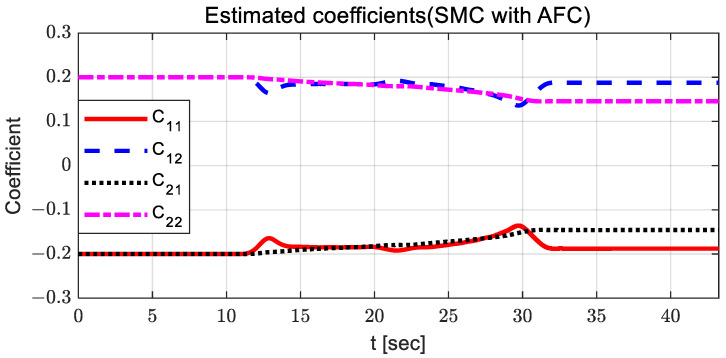
Results: estimated coefficients in the case of SMC with AFC for the curved path tracking.

For AFC, the steering control input is relatively large, and oscillation occurs between 10 and 15 s. The steering control input with SMC has a relatively large value around 23 s, with chattering. When using SMC with AFC, the steering control input is relatively stable compared to other steering control inputs. In the case of PID, the steering control input is relatively high after 23 s, with large oscillations. Figure 6 and Figure 7 show the estimated coefficients for feedback gain adaptation in the cases of AFC and SMC with AFC, respectively.

**Figure 8:**
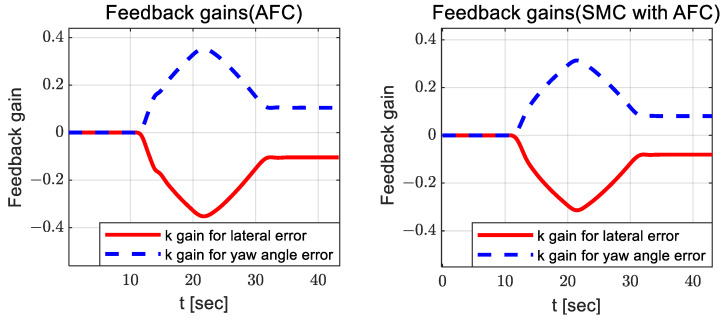
Results: adapted feedback gains (AFC—**left**; SMC with AFC—**right**) for the curved path tracking.

**Figure 9:**
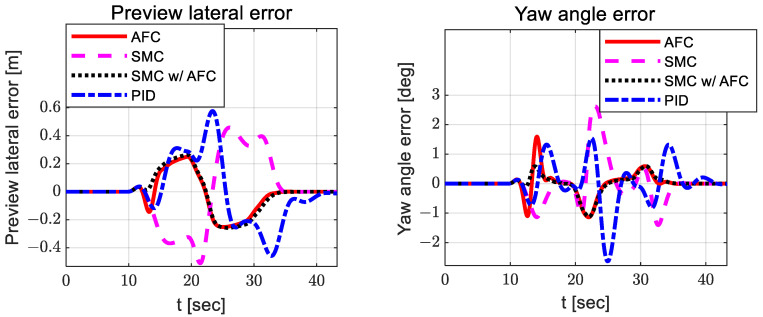
Results: path-tracking control errors (lateral—**left**; yaw angle—**right**) for the curved path tracking.

It can be observed that there is no significant difference between AFC and SMC with AFC; however, the estimated coefficients for SMC with AFC have a relatively small variation around 13 and 30 s. Figure 8 and Figure 9 show the adapted feedback gains and path-tracking control errors (i.e., preview lateral error and yaw angle error), respectively.

**Figure 10:**
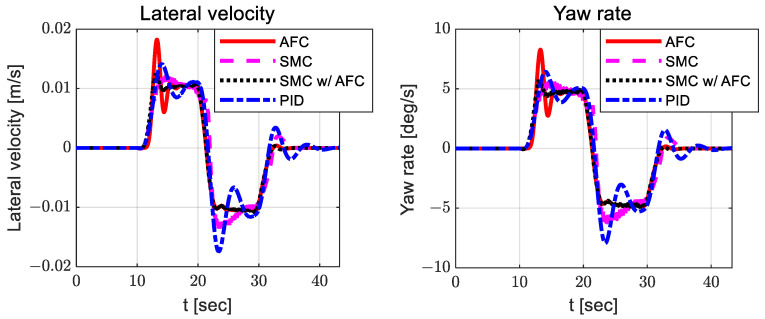
Results: dynamic behaviors (lateral velocity—**left**; yaw rate—**right**) for the curved path tracking.

**Figure 11:**
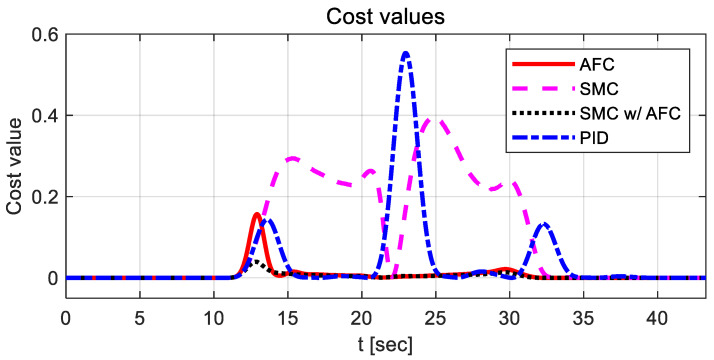
Results: cost value comparison for the curved path tracking.

**Figure 12:**
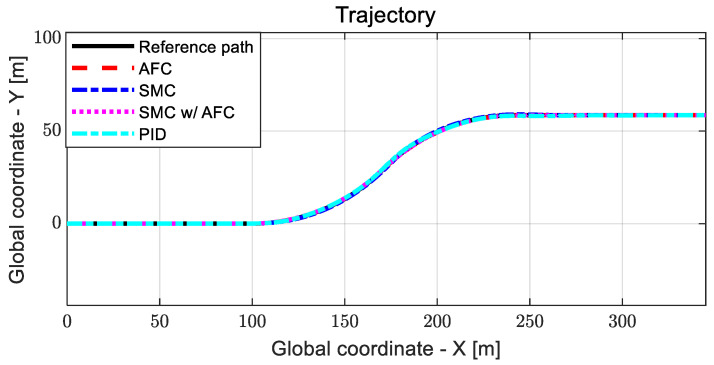
Results: trajectory comparison for the curved path tracking.

According to Figure 8, the adapted feedback gains between AFC and SMC with AFC do not differ significantly, but the feedback gains for SMC with AFC are slightly smaller than those for AFC. Additionally, SMC with AFC shows smaller preview yaw and lateral errors than AFC, SMC, and PID. Figure 10, Figure 11 and Figure 12 show the dynamic behaviors, cost values for path tracking, and vehicle trajectories, respectively.

**Figure 13:**
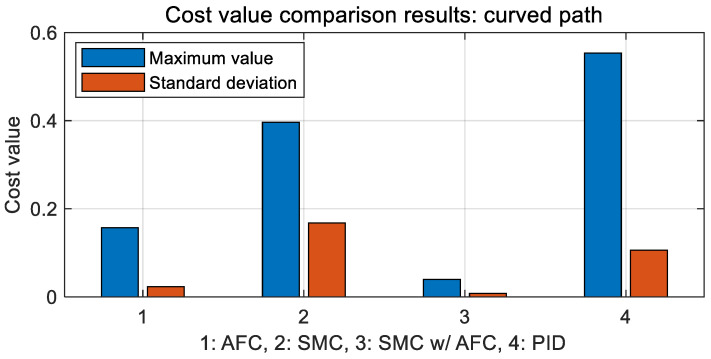
Results: cost value comparison in bar chart form for the curved path tracking.

In Figure 11, PID has the highest cost value for path tracking. There is no significant difference between AFC and SMC with AFC in terms of cost value during the simulation, except for 13 s; however, SMC with AFC shows the smallest value among the three cases. Table 4 and Figure 13 compare the maximum and standard deviations of cost values in each case.

We can note that the maximum and standard deviation values for SMC with AFC are the lowest of all cases. It can also be seen that the SMC-based path-tracking algorithm with adaptive feedback action shows better performance.

### 3.2. Path-Tracking Scenario: Lane Change (60 kph)

**Figure 14:**
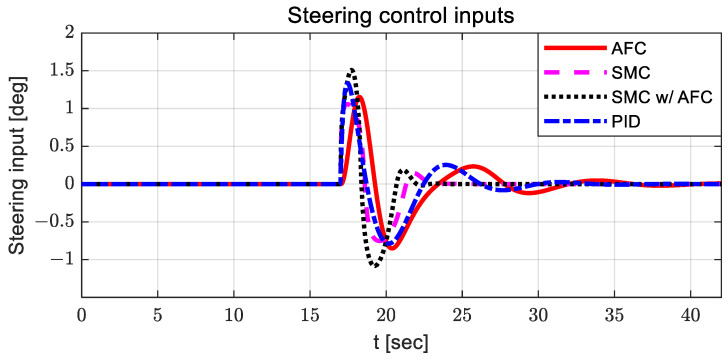
Results: steering control inputs for the lane change.

This section provides performance evaluation results for the lane change scenario with a constant velocity condition of 60 kph. The lane change scenario was designed by switching the desired straight paths so that the vehicle could perform the lane change task reasonably. The time delay function was also used to smooth the path-tracking control errors. Figure 14 illustrates the steering control inputs for the lane change scenario for all cases: AFC, SMC, SMC with AFC, and PID. It can also be observed that the steering control input in the case of SMC with AFC has relatively large values compared to the others. Finally, AFC and PID show some oscillations in the steering control input and slower responses.

**Figure 15:**
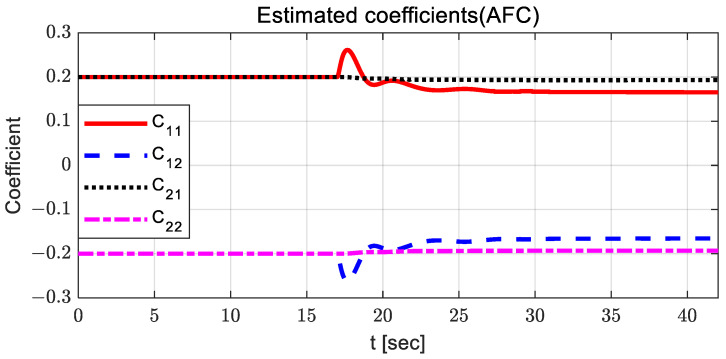
Results: estimated coefficients in the case of AFC for the lane change.

**Figure 16:**
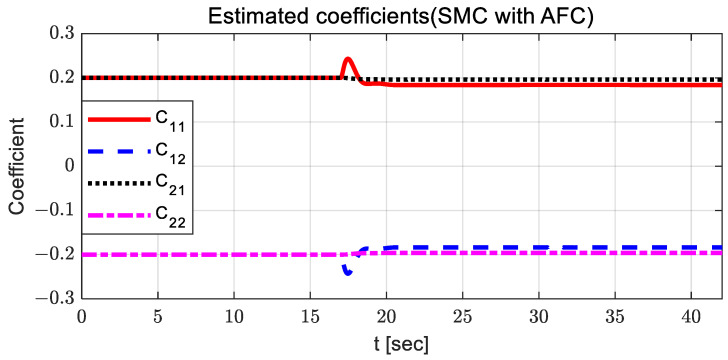
Results: estimated coefficients in the case of SMC with AFC for the lane change.

Figure 15 and Figure 16 show the estimated coefficients for feedback gain adaptation in the cases of AFC and SMC with AFC, respectively.

**Figure 17:**
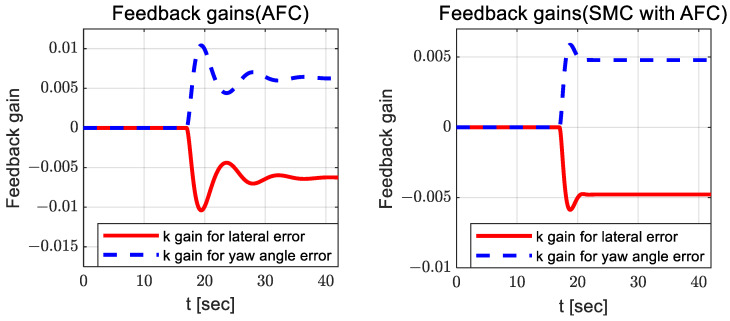
Results: adapted feedback gains (AFC—**left**; SMC with AFC—**right**) for the lane change.

**Figure 18:**
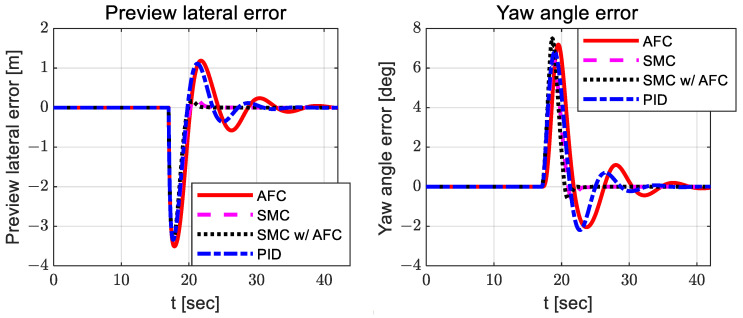
Results: path-tracking control errors (lateral—**left**; yaw angle—**right**) for the lane change.

There are no significant differences between SMC with AFC and AFC in terms of their estimated coefficients and their variation patterns. In the case of using only AFC, there is a relatively larger change in the estimated coefficients because AFC produce steering control inputs for path tracking exclusively, without further assistance from the SMC input. Figure 17 and Figure 18 show the adapted feedback gains and path-tracking control errors, respectively.

In Figure 17, AFC and PID exhibit relatively larger oscillations than SMC with AFC. In Figure 18, there are also similar oscillations in path-tracking control error between SMC and SMC with AFC, but their values are not greatly different. In addition, SMC with AFC shows a higher convergence rate for the preview lateral error and yaw angle error than AFC, SMC, or PID.

With AFC and SMC, the preview lateral error and yaw angle error are more likely to converge than with AFC or SMC alone.

**Figure 19:**
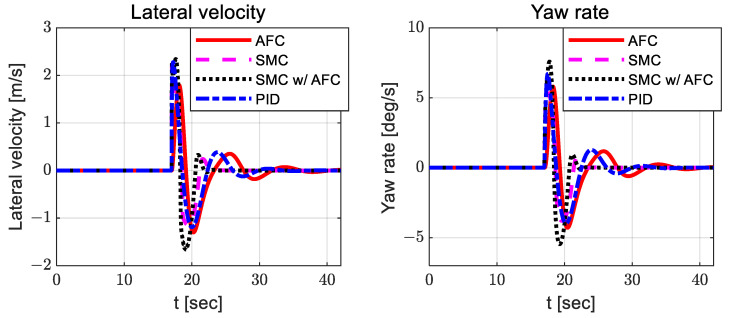
Results: dynamic behaviors (lateral velocity—**left**; yaw rate—**right**) for the lane change.

**Figure 20:**
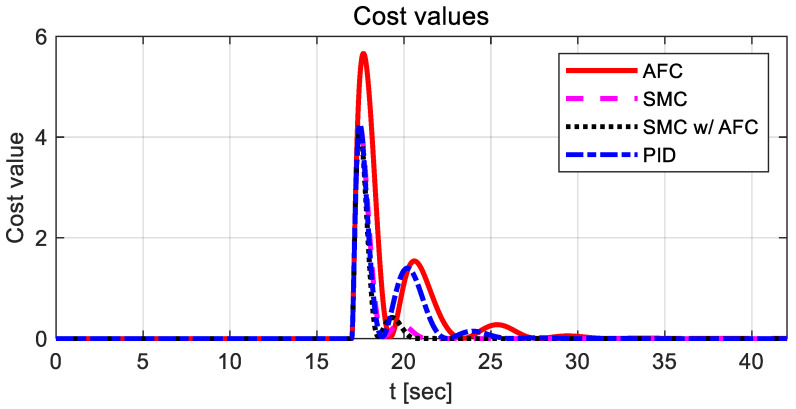
Results: cost value comparison for the lane change.

**Figure 21:**
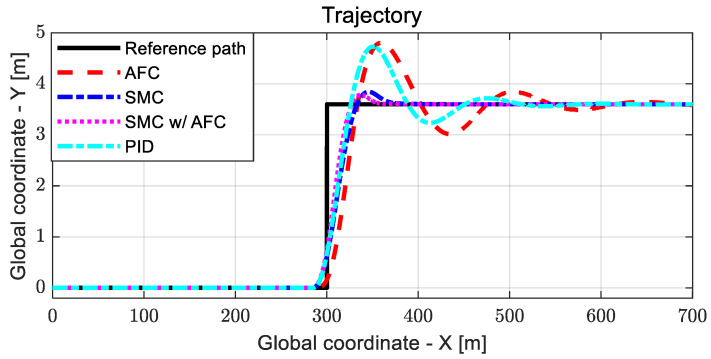
Results: trajectory comparison for the lane change.

Figure 19, Figure 20 and Figure 21 show the dynamic behaviors, cost values for path tracking, and vehicle trajectories, respectively.

**Figure 22:**
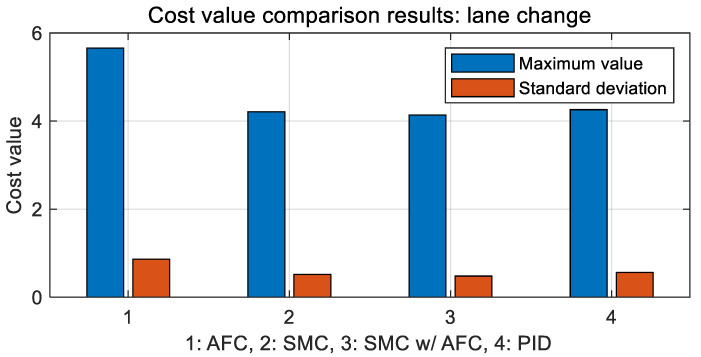
Results: cost value comparison in bar chart form for the lane change.

As shown in Figure 20, AFC has the highest cost value with oscillations for a lane change, while SMC and SMC with AFC show similar variations in cost values. Figure 21 shows the vehicle trajectories for the same lane change. The results indicate that path-tracking control with AFC occurs a little later than the other cases, while showing relatively large overshoots and oscillations. Furthermore, the stabilization rates of the path-tracking controllers using SMC and SMC with AFC are higher than those of AFC alone and PID. In Table 5 and Figure 22, the maximum values and standard deviations of the cost values are compared for each control method.

The above table shows that the maximum and standard deviation values in the case of SMC with AFC are the lowest among the four cases, while they differ slightly for SMC, SMC with AFC, and PID.

Based on the above results, it can be seen that the SMC-based path-tracking algorithm with adaptive feedback action shows reasonable tracking performance under the lane change scenario. In the next section, we discuss this study’s conclusions, limitations, and prospects for future work.

## 4. Conclusions

This study proposes an SMC-based path-tracking control algorithm with adaptive feedback action for autonomous vehicles. The adaptive feedback and SMC algorithms were integrated to enhance the adaptiveness and robustness of the path-tracking control algorithm. The mathematical error model used for the controller design was based on the kinematic mathematical error model. The AFC algorithm was designed using recursive least squares with the forgetting factor and gradient descent methods based on a designed relationship function that uses a combination of path-tracking control errors and feedback gains. Based on the modification of the mathematical error model by the AFC input, the SMC algorithm was designed with finite stability conditions using the Lyapunov theorem. To avoid chattering phenomena and conflict of the SMC input with the AFC input, the sigmoid function was used with proper parameters for gradients. The performance evaluation was conducted under two scenarios (i.e., curved path tracking and lane changes) with constant velocity conditions. The evaluation results show that the control algorithm proposed in this study was able to track the designed reference path reasonably. However, some control parameters should be determined properly for reasonable performance. Therefore, future work will focus on improving the model-free adaptiveness and robustness of the control algorithm. Despite these limitations, it is expected that the developed control algorithm could be widely used for path-tracking algorithms for autonomous vehicles using a simple mathematical model with low computational costs.

## Figures and Tables

**Table 1:** A comparison of the pros and cons of several control methods.

Control Method and Representative Studies	Main Features	Pros	Cons
Proposed	Integrative control using AFC and SMC; a simple model can be used	Adaptive feedback action and robust control considering adaptation are possible	Parameters such as adaptation rate and weighting factor need to be properly determined
Model-based control Refs. [1,2,3]	Optimal control using a system mathematical model	Optimal control allocation is possible	It is necessary to know the system parameters and uncertainty, as well as their rejection
Model-based adaptive control Refs. [20,21,22]	Optimal control with a mathematical model and the adaptation law	Adaptive optimal control is possible	A proper determination of the controller’s adaptation rate is needed for stability
Model-free adaptive control Refs. [23,39,44]	Adaptive control without a system mathematical model	A system mathematical model is not needed	Optimal control allocation is difficult for multi-input systems
Data-driven control Refs. [37,42,46]	Adaptive control and observation using control and system data	Control and observation are possible using only data (without a model)	A stability analysis is required
Learning-based control Refs. [40,48,49]	Control using a learning framework such as reinforcement learning	Performance can be enhanced gradually	To maintain stability, a stability analysis and adaptation of learning rate are required

**Table 2:** Vehicle specification.

Parameter	Unit	Value
Mass	kg	1600
Distance between the front axle and the center of mass	m	1.75
Distance between the rear axle and the center of mass	m	1.20
Wheel tread	m	1.65
Cornering stiffness, front	N/rad	74,000
Cornering stiffness, rear	N/rad	140,000

**Table 3:** Control parameters.

Parameter	Value (Curved Path)	Value (Lane Change)
Forgetting factor	0.999	0.999
Weighting factor (w)	5	5
Coefficient for sigmoid function (m)	1	1
Adaptation gain ($\gamma_{y}$)	1	0.001
Adaptation gain ($\gamma_{\varphi }$)	1	0.001
Parameter for stability condition (α)	1	1
Proportional gain ($k_{p}$)	0.05	0.008
Integral gain ($k_{i}$)	0.02	0.0001
Derivative gain ($k_{d}$)	0.001	0.00001

**Table 4:** Results of cost value comparison for the curved path tracking.

Division	Maximum	Standard Deviation
Adaptive feedback control (AFC)	0.1568	0.0231
Sliding mode control (SMC)	0.3964	0.1678
SMC with AFC	0.0395	0.0078
Proportional–integral–derivative (PID)	0.5535	0.1058

**Table 5:** Results of cost value comparison for the lane change.

Division	Maximum	Standard Deviation
AFC	5.6590	0.8657
SMC	4.2110	0.5197
SMC with AFC	4.1395	0.4816
PID	4.2591	0.5635

## Data Availability

Not applicable.
